# Endoscopic ultrasound-guided pancreatogastrostomy using a novel double lumen dilator

**DOI:** 10.1055/a-2667-7241

**Published:** 2025-09-04

**Authors:** Jun Matsuno, Takeshi Ogura, Takafumi Kanadani, Ahmad Fikry Aboelezz, Hiroki Nishikawa

**Affiliations:** 1 2nd Department of Internal Medicine, Osaka Medical and Pharmaceutical University, Osaka, Japan; 2 13010 Endoscopy Center, Osaka Medical and Pharmaceutical University, Osaka, Japan; 3 68781 Department of Internal Medicine, Gastroenterology and Hepatology Unit, Tanta University, Tanta, Egypt

Endoscopic ultrasound-guided pancreatogastrostomy (EUS-PG) can be indicated for patients with pancreatic obstruction after a failed endoscopic retrograde cholangiopancreatography (ERCP) 1 2 3 . The technical steps of EUS-PG include pancreatic duct puncture, guidewire deployment, tract dilation, and stent deployment. Notably, compared with the EUS-guided transhepatic approach, the echoendoscope is not stable because its upper angle is not as strong during EUS-PG. During device insertion, such as stent deployment, this instability may lead to inadequate axis and cause performing EUS-PG to be more challenging.

To overcome this problem, a novel double-lumen dilation device (Meissa, Japan Life Line, Tokyo, Japan) was developed ( Fig. 1 ). This device has a 2.3-Fr tip and a maximum diameter of 7.4 Fr, with a 2-cm side hole provided from the tip that allows for contrast medium injection, aspiration of the pancreatic juice, and 0.025-in. guidewire insertion. As such, this device can be used to perform the double-guidewire technique without additional device exchange using a 0.018-in. guidewire. Herein, we describe the technical tips for performing EUS-PG using this novel dilator.

**Fig. 1:**
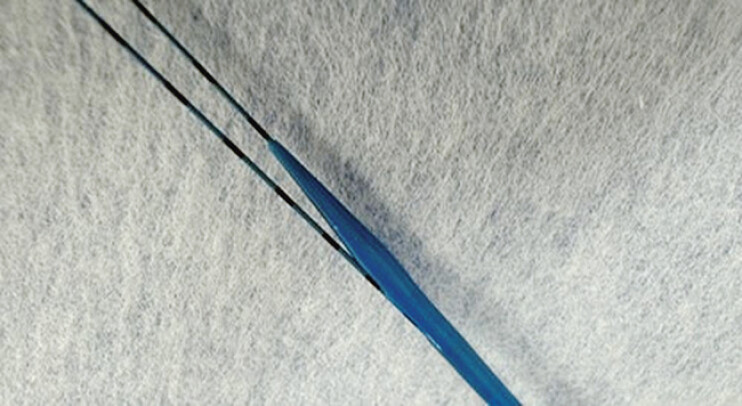
Novel double-lumen dilation device (Meissa; Japan Life Line, Tokyo, Japan).

A 78-year-old woman with recurrent pancreatitis due to a pancreatic stone was referred to our hospital after a failed ERCP-guided pancreatic stent deployment at another hospital. Consequently, an EUS-PG was attempted. The pancreatic duct was punctured using a 19-G needle, and contrast medium was injected. A 0.025-in. guidewire was then inserted ( Fig. 2 ). Next, the novel dilation device was inserted into the pancreatic tract ( Fig. 3 ). A 0.025-in. guidewire was inserted through the side hole of the novel dilator ( Fig. 4 ). After tract dilation, a 7.0-Fr stent was easily inserted and successfully deployed from the pancreatic duct to the stomach ( Fig. 5 ) without any adverse events ( Video 1 ).

**Fig. 2:**
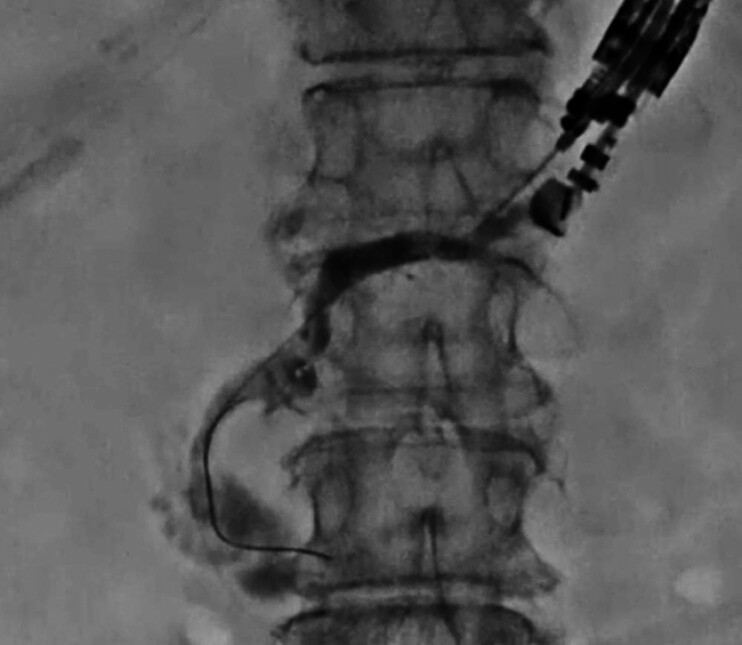
The pancreatic duct is punctured; after contrast injection, the guidewire is advanced into the duodenal lumen.

**Fig. 3:**
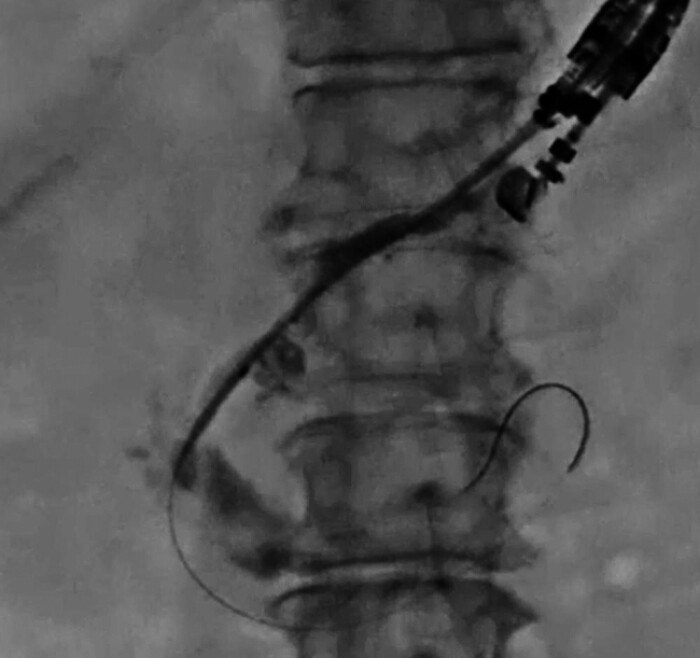
The pancreatic and gastric walls are dilated using a novel dilator, and the side hole of the novel dilator is advanced into the pancreatic duct.

**Fig. 4:**
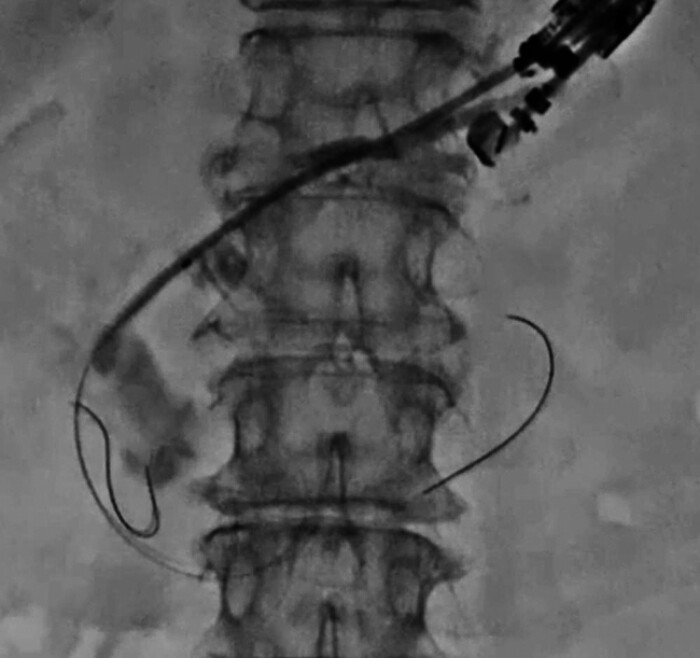
A 0.025-in. guidewire is inserted through the side hole of the novel dilator.

**Fig. 5:**
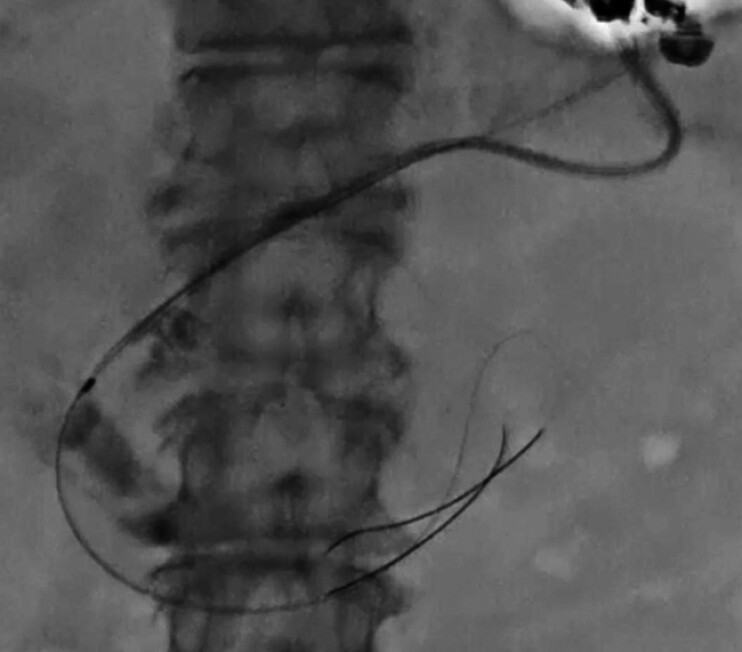
After tract dilation, a 7.0-Fr stent is easily inserted and successfully deployed from the pancreatic duct into the stomach.

A novel double-lumen dilator is inserted, and a 7.0-Fr pancreatic stent is deployed.Video 1

In conclusion, this dilation device allows the double-guidewire technique without the need for additional device exchanges and may be useful for EUS-PG.

Endoscopy_UCTN_Code_TTT_1AS_2AD

## Notes

Endoscopy E-Videos https://eref.thieme.de/e-videos
*E-Videos* is an open access online section of the journal *Endoscopy* , reporting on interesting cases and new techniques in gastroenterological endoscopy. All papers include a high-quality video and are published with a Creative Commons CC-BY license. Endoscopy E-Videos qualify for HINARI discounts and waivers and eligibility is automatically checked during the submission process. We grant 100% waivers to articles whose corresponding authors are based in Group A countries and 50% waivers to those who are based in Group B countries as classified by Research4Life (see: https://www.research4life.org/access/eligibility/ ). This section has its own submission website at https://mc.manuscriptcentral.com/e-videos .
